# Motivating residents to change communication: the role of a brief motivational interviewing didactic

**DOI:** 10.1017/S146342361900015X

**Published:** 2019-08-27

**Authors:** Lisa Renee Miller-Matero, Erin T. Tobin, Elizabeth Fleagle, Joseph P. Coleman, Anupama Nair

**Affiliations:** 1 Internal Medicine, Henry Ford Health System, Detroit, MI, USA; 2 Behavioral Health, Henry Ford Health System, Detroit, MI, USA; 3 Center for Health Policy and Health Services Research, Henry Ford Health System, Detroit, MI, USA

**Keywords:** medical education, motivational interviewing, primary care

## Abstract

**Introduction:**

Motivational interviewing (MI) is a patient-centered approach that encourages patients to change behaviors. MI training programs have increased residents’ knowledge and use of MI skills; however, many residency programs may not have the time to dedicate to lengthy MI programs. The purpose of this study was to evaluate the benefits of a brief MI didactic for residents in an academic internal medicine patient-centered medical home.

**Methods:**

Thirty-two residents completed a 1-h MI training between October 2016 and June 2017 and completed measures on their knowledge of, confidence using, and utilization of MI skills prior to the training, immediately after the training, and at a 1-month follow-up.

**Results:**

The residents’ knowledge of and confidence using MI skills increased from pre-test to post-test and also increased from pre-test to the 1-month follow-up.

**Conclusion:**

The utilization of MI skills increased from pre-test to the 1-month follow-up. A 1-h didactic offers benefits to residents.

## Introduction

Motivational interviewing (MI) is a patient-centered counseling approach aimed at eliciting behavior change in patients (Rollnick and Miller, 1995). It places emphasis on provider empathy and collaboration with patients to enhance patient self-efficacy and motivation in decision-making (Rollnick and Miller, 1995; Rollnick, 2002). Research has demonstrated that the use of MI skills provides clinically significant benefits within patient care for a range of issues, including substance use, poor health behaviors (e.g., diet, exercise), or difficulty with treatment adherence (Hettema *et al*., 2005; Vasilaki *et al*., 2006; Lundahl and Burke, 2009). Although many providers see the importance of counseling patients regarding health behavior change and prevention efforts, few provide this care (Yeager *et al*., 1995), and many providers feel unprepared to do so (Jallinoja *et al*., 2007; Midboe *et al*., 2011). The literature has also demonstrated that physician’s use of MI skills can improve patient interactions (Cucciare *et al*., 2012) and that training is associated with less physician burnout and higher personal accomplishment (Pollak *et al*., 2016). Thus, learning MI skills early in residency training may improve not only patient care and outcomes but also physician health and wellness.

MI training has been conducted across a number of medical residency training programs and specialties for physicians post their medical school training and who are undergoing supervised practice. These training programs have increased residents’ knowledge about MI, their MI skill level, use of MI skills, and their confidence in using these skills immediately after training (Scal *et al*., 2004; Johns *et al*., 2010; Lozano *et al*., 2010; Yu and Beresford, 2010; Childers *et al*., 2012; Cole *et al*., 2012; Dunhill *et al*., 2014). Residents’ beliefs about the importance of using MI skills also increased post training (Childers *et al*., 2012). The majority of training programs use pre- and post-tests to measure training effectiveness (Dunhill *et al*., 2014). Few studies that have included evaluations at least 1 month post training reported varying degrees of effectiveness (Scal *et al*., 2004; Lozano *et al*., 2010). For example, although there was improvement in MI spirit (i.e., embracing the ideology of MI) and MI skills several months post training in one study, another study found no change in residents’ views of importance of and effectiveness of MI despite the use of a booster session 6 weeks later (Scal *et al*., 2004; Lozano *et al*., 2010). Therefore, additional research is needed to evaluate whether there are longer-term benefits of teaching MI to medical residents.

Much of the literature on MI training includes lengthy training programs, while only a few of these institute shorter training models (Lozano *et al*., 2010; Yu and Beresford, 2010; Burton *et al*., 2011; Childers *et al*., 2012). MI programs range from 2.5- to 12-h-long workshops (Johns *et al*., 2010; Childers *et al*., 2012). At this time, it is not clear that longer MI training programs produce better outcomes than shorter programs (Cole *et al*., 2012). Although the importance of MI training is recognized by many (Dunhill *et al*., 2014), relatively little time is typically committed to MI training for medical residents (Lazare and Moaveni, 2016). Given that many residency programs likely do not have the time to devote to lengthy MI training, it is possible that providing very brief MI training may offer benefits both at post training and a longer-term follow-up. However, the existing studies that have examined improvements weeks or months post training have not done so in the context of brief training (Scal *et al*., 2004; Lozano *et al*., 2010).

The primary objective of this study was to evaluate the benefits of a brief, 1-h didactic on MI with internal medicine residents post their medical school training and in supervised practice. It was hypothesized that residents’ knowledge and confidence regarding MI strategies would increase from pre- to post teaching and that knowledge, confidence, and utilization of MI strategies would increase from baseline to a 1-month follow-up.

## Methods

### Participants and procedure

Thirty-two residents in an academic internal medicine patient-centered medical home at an urban hospital participated in the current study. Residents participated in this MI training during a rotation in which they spend a month in the academic internal medicine primary care clinic. Each month, three or four residents, postgraduate years 1 or 2 in supervised training, complete this rotation. Participants in the current study completed the rotation between October 2016 and June 2017.

During the first week of the internal medicine primary care clinic rotation, the staff psychologist and psychology trainees provided a 1-h didactic on the fundamentals of MI. Residents were asked to complete a pre-test regarding their knowledge of MI, confidence of using MI, and utilization of MI. After the 1-h teaching, residents completed a post-test on their knowledge of and confidence in using MI. Approximately 1 month after the teaching, residents were asked to complete the same measures addressing knowledge, confidence, and utilization of MI skills. The 1-month follow-up measures were completed by 65.6% (*n* = 21) of the participants. This study was approved by the institution’s research review board.

### Materials

Given the brevity of training provided, we developed brief measures to assess MI knowledge, confidence in using MI in patient care, and utilization of MI skills.

MI knowledge was measured by the completion of two multiple-choice and two open-ended questions regarding the spirit, principles, and fundamentals of MI. Multiple-choice questions included items assessing residents’ knowledge of what MI is and the spirit of MI (Table 1). Open-ended questions asked the residents to list the four primary principles of MI and four fundamental MI skills. Primary principles counted as correct included resisting the righting reflex, understanding patient motivation, listening with empathy, and empowering the patient. The four fundamental MI skills counted as correct included: open-ended questions, affirmations, reflections, and summary statements.

**Table 1.** Residents’ knowledge of and confidence in using motivational interviewing skills

Knowledge questions	Pre-test, *M* (SD)	Post-test, *M* (SD)^a^	*t*	*P*	Follow-up, *M* (SD)^a^	*t*	*P*
Motivational interviewing is a ____________, practitioner-directed method for enhancing ___________ motivation to change by exploring and resolving ambivalence. (1 point) consequence-driven; low superior; non-existent famous; outward client-centered; intrinsicAnswer: D	0.97 (0.18)	1.00 (0.00)	−1.00	0.33	1.00 (0.00)	−1.00	0.33
Which of the following words is not used to describe the spirit of motivational interviewing? (1 point) autonomy expertise evocation collaborationAnswer: B	0.66 (0.48)	0.94 (0.25)	−3.48	0.002**	0.94 (0.24)	−2.56	0.02*
3. What are the four primary principles in MI? (4 points) Answer: Resisting the righting reflex; understanding patient motivation; listening with empathy; empowering the patient	0.09 (0.30)	1.97 (1.38)	−7.93	<0.001**	2.17 (1.20)	−7.51	<0.001**
4. What are the four fundamental MI skills? (4 points)Answer: open-ended questions; affirmations; reflections; summary statements	0.22 (0.61)	3.22 (1.13)	−13.64	<0.001**	2.61 (1.58)	−5.24	<0.001**
Total score (10 points)	1.94 (0.95)	7.13 (2.01)	−13.57	<0.001**	6.72 (2.35)	−6.70	<0.001**
Confidence questions	Pre-test, *M* (SD)	Post-test, *M* (SD)^a^	*t*	*P*	Follow-up, *M* (SD)^a^	*t*	*P*
1. On a scale of 0 (not confident at all) to 10 (complete confidence), please indicate your level of confidence in building and strengthening your patients’ motivation to engage in lifestyle change.	5.33 (1.65)	6.83 (1.24)	−6.86	<0.001**	6.89 (1.71)	−2.81	0.01*
2. On a scale of 0 (not confident at all) to 10 (complete confidence), please indicate your level of confidence in using motivational interviewing skills.	4.72 (1.76)	6.72 (1.25)	−9.09	<0.001**	6.72 (1.71)	−4.19	0.001**
Total score (20 points)	10.05 (3.16)	13.55 (2.40)	−8.76	<0.001**	13.61 (3.27)	−3.96	0.001**

a Compared with pre-test scores.

* *P* < 0.05.

** *P* < 0.01.

To assess residents’ confidence in using MI skills, they were asked to complete two items (Table 1). Specifically, residents were asked to rate their confidence in building and strengthening patients’ motivation to engage in lifestyle changes on a 11-point Likert scale ranging from 0 (not confident at all) to 10 (complete confidence). Residents were also asked to rate their confidence in using motivational interviewing skills on a 11-point Likert scale ranging from not confident at all (0) to complete confidence (10).

**Table 2.** Residents’ utilization of motivational interviewing skills

Question	Pre-test, *M* (SD)	Follow-up, *M* (SD)	*t*	*P*
1. How often do you find yourself having the desire to fix your patients by telling them what to do?^a^	2.10 (0.70)	2.62 (0.97)	−2.75	0.01*
2. How often do you ask your patients about their motivation to change unhealthy behaviors	3.24 (0.77)	3.67 (0.58)	−2.26	0.04*
3. How often do you use open-ended questions in your clinical practice?	4.29 (0.46)	4.33 (0.58)	−0.33	0.75
4. How often do you make statements that recognize patients’ strengths?	3.57 (0.75)	3.62 (0.74)	−0.19	0.85
5. How often do you use simple reflective listening, rephrasing what patients say?	3.76 (0.63)	3.71 (0.64)	0.30	0.77
6. How often do you use complex reflections to emphasize emotions associated with patients’ statements?	3.00 (0.95)	3.38 (0.92)	−1.90	0.07
7. How often do you use summaries with your patients during appointments?	4.00 (0.95)	3.90 (0.54)	0.40	0.69
**Total score (35 points)**	23.95 (2.26)	25.24 (2.76)	−2.42	0.03*

Note. Answers were on a 1–5 scale from never to always.

a Item was reverse-scored.

* *P* < 0.05.

MI utilization was assessed using a seven-item scale displayed in Table 2. Residents were asked to respond on a five-point Likert scale ranging from never (1) to always (5).

### Analyses

Means and standard deviations of items from the measures at pre-test, post-test, and 1-month follow-up are included in Tables 1–2. Paired samples *t*-tests were conducted to compare mean differences between pre- and post-test measures of MI knowledge and confidence in using motivational interviewing in patient care. Paired samples *t*-tests were also conducted to compare mean differences between pre-test and 1-month follow-up data regarding MI knowledge, confidence, and utilization.

## Results

The residents’ knowledge of MI skills increased from pre- to post-test and also increased from pre-test to 1-month follow-up (Table 1). The residents’ confidence in using MI skills increased from pre- to post-test and also increased from pre-test to 1-month follow-up (Table 1). Finally, the utilization of some of the MI skills increased from pre-test to 1-month follow-up (Table 2).

## Discussion

The purpose of the present study was to evaluate the potential benefits of a brief MI didactic. Results suggest that a brief MI didactic is an effective way to teach MI skills. Not only did residents’ knowledge and confidence of utilizing MI skills increase from baseline to post-test, this increase was maintained at 1-month follow-up. In addition, residents’ knowledge, confidence, and utilization of MI skills increased from baseline to 1-month follow-up. These findings are consistent with other studies that have examined MI training in graduate medical education (e.g., Dunhill *et al*., 2014).

The brevity of the intervention is perhaps the most unique aspect of this training. The training in the current study consists of only a 1-h didactic on MI fundamentals, whereas previous trainings were at least 2.5 h in length (Dunhill *et al*., 2014). Although these interventions are correlated with favorable outcomes for residents’ use of MI skills and acceptance and/or confidence of the MI approach, the large time allotment may not be feasible in all settings. The present study suggests that even a 1-h didactic is associated with improvements in knowledge and confidence post-test, and knowledge, use, and confidence at a 1-month-follow up.

Although statistically significant improvement in total utilization scores was seen between baseline and follow-up, item analysis revealed a more complex picture. It appeared that the greatest change between baseline and follow-up was from the item assessing resident’s desire to “fix” their patients. This desire to “fix” may place undue feelings of responsibility on residents, and may lead to increased burden. A recent study of a staff-wide MI training found improvements in clinician burnout (Pollak *et al*., 2016). No studies examining physician resident burnout in relation to MI were found; however, this may be particularly relevant given the high rates of burnout in medical trainees and clinicians (Dyrbye *et al*., 2014). Future studies could evaluate the relationship between MI training and burnout in this population.

There are some limitations of the present study. There was attrition for the 1-month follow-up. It is possible that those who did not complete the 1-month follow-up had lower rates of MI knowledge, use, and confidence, which is consistent with a meta-analysis on MI skill maintenance indicating skill erosion was associated with attrition rates (Schwalbe *et al*., 2014). In addition, the outcome measure of utilization in the present study was self-report. There may be a discrepancy between perceived utilization and actual utilization. Future studies should evaluate objectively whether this education increases the use of motivational interviewing strategies in practice. Furthermore, outcome measures were created with the purpose of the present study in mind. Although based on the foundation and spirit of MI, these have yet to be validated. Future research could explore more meaningful outcomes of providers’ observed use in either a writing task (e.g., Helpful Responses Questionnaire; Miller *et al*., 1991) or clinical situation (e.g., observed clinical encounters scored via Motivational Interviewing Treatment Integrity Code; Moyers *et al*., 2016).

In the present study, residents were assessed immediately following teaching and 1 month later. Future research could evaluate whether these changes are sustained for a period >1 month. Meta-analytic review has found skill erosion at 6-month follow-up in the absence of intermittent ongoing training (Schwalbe *et al*., 2014). In addition, booster sessions have been shown to improve proficiency of MI in primary care clinicians, with statistically significant improvements in observed skills, confidence, and knowledge (Fu *et al*., 2015). A longer period of follow-up is warranted as is incorporating additional training in a booster session model. Future research could also evaluate whether patient outcomes improve after residents participate in MI training.

In conclusion, the present study suggests that a brief, 1-h MI training can improve resident knowledge, confidence, and use of MI skills at 1-month follow-up. These preliminary findings suggest that brief interventions may be a way to incorporate MI education into graduate medical education, especially when longer trainings are not feasible. Future studies addressing brief MI training modalities using longer periods of follow-up, assessing booster sessions, and incorporating objective outcomes are warranted.
